# Radium’s Place in Medicine

**Published:** 1904-02

**Authors:** 


### EDITORIAL NOTES AND COMMENTS.

The Business office of THE JOURNAL-RECORD is No. 1007-1008 Century Building.

The Editorial office is 318-319-320 The Prudential.

Address all Business communications to Dr. M. B. Hutchins, Mgr.

Make remittances payable to THE ATLANTA JOURNAL-RECORD OF MEDICINE.

On matters pertaining to the Editorial and Original Communications address Dr Bernard Wolff, Atlanta.

Reprints of original articles will be furnished at cost price. Requests for the same should always be made *on the manuscript.*

We will present, post-paid, on request, to each contributor of an original article, twenty (20) marked copies of THE JOURNAL-RECORD containing such article.

### RADIUM'S PLACE IN MEDICINE.

THE wonderful substance, radium, which seems to set at naught so many preconceived natural laws, has been subjected to experiments in the field of therapeutics. A substance so energetic when properly curbed was judged to have effect in the treatment of somewhat similar diseased conditions to those which the X-rays are applied. The almost prohibitive price of such salts of radium as are available for clinical purposes have rendered such use of it very limited. A certain amount of work, however, has been done and the results published. The most favorable report of its effect in cancer (one of its first therapeutic applications) was from Vienna. There two cases of inoperable recurrent cancer were reported as having been cured by bromide of barium. Dr. Cleaves, of New York, found that a case of sarcoma of the jaw, and one of carcinoma of the uterus had been benefited by radium. She used a tube of 1500 and one of 7000 activity. The degree of activity is expressed in relation to electric conductivity of the air as compared with uranium as the standard. Danloz, of the Pasteur Institute at Paris, has given out some guarded expressions as to the utility of radium in cancer therapy.

In England the results appear to be in the main negative, though some instances of apparent healing of epithelioma have been recorded.

The discovery in Utah and Texas of uranirite earth or pitchblende from which radium may be got and the efforts made to simplify and cheapen the cumbersome and slow present method of extraction of radium may serve to put it in a position to be more widely used. More will then be known of it as a therapeutic agent.

The effect seems comparable to that of the X-ray but without the electric element. It produces extensive burns which are slow to heal, and causes the hair to fall out. It has a paralysant effect upon small animals when they are exposed to the radiance for any length of time.

The revelations of the future, especially in regard to the application of radium as a remedy, will be awaited with interest, but at present its position seems too untried to warrant the expense of using it for clinical purposes.

